# The association of age with decline in renal function after low anterior resection and loop ileostomy for rectal cancer: a retrospective cohort prognostic factor study

**DOI:** 10.1186/s12877-020-02001-z

**Published:** 2021-01-19

**Authors:** Amal Rhemouga, Stefan Buettner, Wolf O. Bechstein, Guido Woeste, Teresa Schreckenbach

**Affiliations:** 1 Department of General, Visceral and Transplantation Surgery, Frankfurt University Hospital and Clinics, Goethe-University Frankfurt/Main, Theodor-Stern-Kai 7, 60596 Frankfurt/Main, Germany; 2 Department of Nephrology, Frankfurt University Hospital and Clinics, Goethe-University Frankfurt/Main, Theodor-Stern-Kai 7, 60596 Frankfurt/Main, Germany; 3 Department of General and Visceral Surgery, AGAPLESION Elisabethenstift, Landgraf-Georg-Str. 100, 64287 Darmstadt, Germany

**Keywords:** Rectal cancer, Ileostomy, Older patient, Estimated glomerular filtration rate, Chronic kidney disease

## Abstract

**Background:**

Low anterior resection (LAR) is often performed with diverting loop ileostomy (DLI) for anastomotic protection in patients with rectal cancer. We aim to analyze, if older patients are more prone to a decline in kidney function following creation and closure of DLI after LAR for rectal carcinoma versus younger patients.

**Methods:**

A retrospective cohort study from a database including 151 patients undergoing LAR for rectal carcinoma with DLI was used. Patients were divided in two age groups (Group A: <65 years, *n* = 79; Group B: ≥65 years, *n* = 72). For 123 patients undergoing DLI reversal prognostic factors for an impairment of serum creatinine (SCr) and estimated glomerular filtration rate (eGFR) 3 months after DLI reversal was analyzed using a multivariate linear regression analysis.

**Results:**

SCr before LAR(T_0_) was significant higher in Group B (*P* = 0.04). Accordingly, the eGFR at T_0_ in group B was significantly lower (*P* < 0.001). No patients need to undergo hemodialysis after LAR or DLI reversal.

Age and SCr at T_0_were able to statistically significant predict an increase in SCr (*P*<0.001) and eGFR (*P*=0.001) three months after DLI reversal (The R² for the overall model was .82 (adjusted R² = .68).

**Conclusion:**

DLI creation may result in a reduction of eGFR in older patients 3 months after DLI closure. Apart from this, patients do not have a higher morbidity after creation and closure of DLI resulting from LAR regardless of their age.

.

## Background

Low anterior resection (LAR) for rectal carcinoma is one reason for creation of diverting loop ileostomy (DLI) in older patients. Although DLI does not prevent anastomotic leakage, it does reduce septic complications of leakage, the risk of re-operation, and life-threatening complications [1, 2]. The use of a DLI is believed to be important by many colon and rectal surgeons, as the rate of anastomotic leakage in LAR can be up to 28% without stoma creation [1, 3]. DLI is normally performed assuming that reversal can occur quickly [4]. However, temporary ostomies also negatively influence the patient’s quality of life and can result in stoma-related complications such as acute kidney injury (AKI) due to postoperative complications or dehydration [5, 6].

Patients often fear maintaining a permanent stoma, and surgeons especially worry about the development of a high-output ileostomy followed by serious kidney dysfunction [7]. The readmission rate after performing a DLI is up to 30%, and most readmissions are associated with dehydration [5, 8]. This rate could be even higher in patients with an already impaired kidney function [9]. In addition, reversal surgery for DLI is associated with up to a 20% risk of postoperative complications [4, 10]. Therefore, the routine use of DLI for LAR has been questioned [11].

In this retrospective cohort study, we aim to analyze if older patients are more prone to a decline in kidney function following creation and closure of DLI after LAR for rectal carcinoma versus younger patients.

We hypothesize, that an increasing age is a signifiant prognostic factor for a increase of serum creatine (SCr) and therefore for a decreasing of estimated glomerular filtration rate (eGFR) in patients undergoing LAR with creation of DLI.

## Methods

This study is a retrospective cohort-study performed at a single tertiary care center. The study was conducted in accordance with the Declaration of Helsinki (Ethical Principles for Medical Research Involving Human Subjects) and was approved by the Ethics Committee of the Goethe University, Frankfurt, Germany (no. 435/14).

### Patient demographics and clinical data

**Fig. 1:**
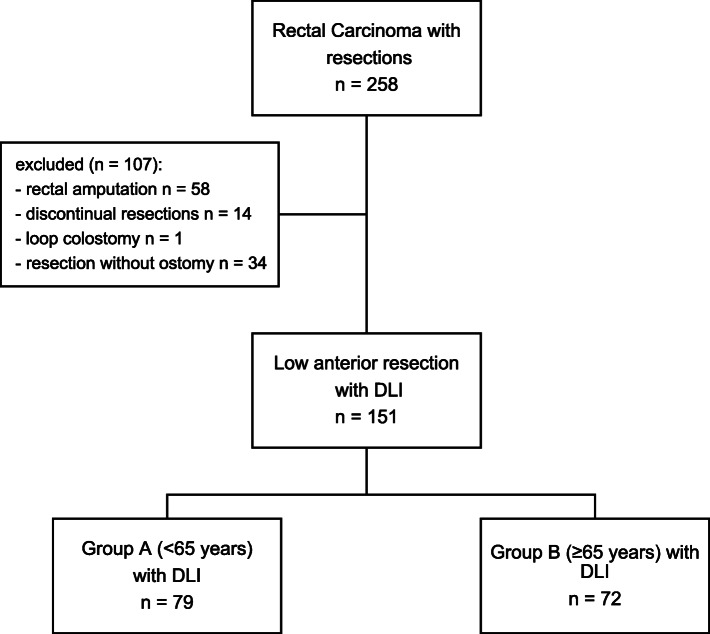
Patient disposition flow chart

We have included patients who underwent rectal resection due to a rectal carcinoma between January 2003 and December 2013. The exclusion criteria were abdominoperineal rectal resection (APR), Hartmann’s procedure, resection with loop colostomy, and resection without stoma (Fig. 1). All patients underwent LAR with DLI. Patient characteristics were collected from the electronic health records and a dedicated University Cancer Center (UCT) database. We used international Classification of disease (ICD), 10th revision, german modification and Operation and Procedure Classification System (OPS) for identification of patients, meeting the inclusion criteria. The data collection was done by one member of the research group and was double-checked by one of the senior authors.

Patients and tumor characteristics were obtained from the database including demographic characteristics, physical status classification according to the American Society of Anesthesiologists (ASA), and initial tumor stage according to the Union for International Cancer Control (UICC; version 2010). Comorbidities included coronary heart disease, chronic obstructive pulmonary disease (COPD), chronic kidney disease (CKD), and diabetes mellitus.

SCr in mg/dl at admission day at the hospital, seven days after the surgery and 3 months after reversal of DLI were collected.

### Definitions

Neoadjuvant treatment was defined as chemotherapy or radiotherapy before tumor resection. Adjuvant therapy was defined as any chemotherapy or radiotherapy after rectal resection. Adjuvant chemotherapy includes 5-fluorouracil or oxaliplatin, folinic acid, and 5-fluorouracil (FOLFOX) or folinic acid, 5-fluorouracil, and irinotecan (FOLFIRI), and capecitabine. Radiotherapy regimes were classical 50.4 Gy radiotherapy or 5 × 5 Gy radiotherapy [12].

For analyzing the influence of age on the decline in kidney function 3 months after DLI reversal, we extracted the SCr at the two different time points: (1) admission day to hospital before LAR (T_0_) and (2) 3 months after DLI reversal (T_1_). SCr samples on T_0_ was defined as baseline SCr.

At both time points, eGFR was calculated from the database using the Chronic Kidney Disease Epidemiology Study (CKI-EPI) algorithm [13]. CKD was graded using the Kidney Disease Improving Global Outcomes guidelines (KDIGO) via the following five stages [14]: grade 1, eGFR ≥ 90 ml/min; grade 2, eGFR 60–89 ml/min; grade 3, eGFR 30–59 ml/min; grade 4, eGFR 15–29 ml/min; and grade 5, < 15 ml/min.

For calculation of postoperative Acute Kidney injury (AKI) we used the highest SCr during the first seven postoperative days (POD) according to the KDIGO-criteria: stage 1, increased SCr x 1.5–1.9 within the preceding 7 days or an SCr increase ≥ 0.3 mg/dl within 48 h; stage II, increased SCr x 2-2.9; stage III, increased SCr x 3 or SCr ≥ 4 mg/dl or initiation of renal replacement therapy (RRT).

### Surgical procedures and postoperative complications

All operation notes were closely scrutinized for whether a LAR was performed or not. LAR was performed as total mesorectal excisions (TME) by an experienced surgical team under the supervision of a board-certified colorectal surgeon at a certified colorectal cancer center. LAR was performed as an open or laparoscopic procedure. The anastomosis types were end-to-end, side-to-end, J-Pouch, and coloanal anastomosis depending on the performing surgeon and the location of the rectal tumor. The anastomoses were performed using a circular stapler except for coloanal anastomosis. The ileostomy side was marked by a ostomy nurse before surgery.

DLI closure was performed as a functional end-to-end anastomosis using a running suture or as a side-to-side anastomosis using a stapler device depending on the surgeon’s preference. The DLI side was closed using interrupted sutures in the earlier years and was later switched to a purse-string closure with a remaining drainage opening in the middle [15]. The aim was to close DLI within four months of LAR.

Postoperative complications and re-interventions after LAR and DLI reversal were collected. Postoperative complications were reviewed and graded using the Clavien–Dindo classification system [16]. Minor complications were categorized as either grade 1 or 2 while major complications were categorized as grade 3 or higher.

### Study endpoints

The primary endpoint of the study were SCr increase and eGFR decrease at T_1_.

### Statistics

All statistical analyses were performed using Statistical Package for the Social Sciences (SPSS) for Windows (version 22.0; IBM, Chicago, IL, USA).

For the descriptive analyzes patients age was dichotomized at age 65 years: Group A (< 65 years) and Group B (≥ 65 years). We defined this age threshold according to a recent review by Hewitt et al., showing that the most included high quality studies used a cut-off of 65 years to define the older population because the incidence of frailty in the patient group that is ≥ 65 years old increased by 25% and has the highest incidence of all age groups [17].

Categorical variables are presented as frequencies and percentages. Continuous variables are presented as the mean and standard deviation (SD). Categorical variables were compared using the chi-squared (χ^2^)-test or Fisher’s exact test as needed. Pearson’s chi-square, the degrees of freedom (df), and Cramer’s *V* as parameter for effect size are shown in all tables. Continuous variables were compared using a 2-sample Student’s *t*-test with Bonferroni correction. *P*-values were derived from two-tailed tests and *P* < 0.05 was considered to be statistically significant for all tests.

Multivariate linear regression analyzes were performed for SCr and eGFR at T_1_ using a forward selection model. Only patients undergoing DLI reversal were included. The factors included in the model were selected according to known risk factors for postoperative kidney failure from current studies [18, 19]. We included patient-related and procedure-related factors. Patient-related factors were age as a continuous parameter, sex, Diabetes mellitus, Hypertension, COPD, ASA score, neoadjuvant and adjuvant chemotherapy, SCr at T_0_, and eGF at T_1_. Procedure-related factors were CDC after LAR and DLI reversal, blood transfusion after LAR, operation time for LAR and time between LAR and DLI reversal. Multivariate regression coefficient, Beta, 95% CI and *P*-value are shown in the results. *P*-values < 0.05 were considered to be statistically significant. For interpretation of the results the F-distribution (F-ratio) as test of overall fit is given in the tables. The results are given in the text in the following form: F-test[F](df, residual) = F-ratio. As measurement of the effect size R^2^ and adjusted R^2^ were used. According to Cohen, R^2^ and adjusted R^2^ can be interpreted as following: strong effect size, R^2^ = 0.26; median effect size, R^2^ = 0.13; weak effect size, R^2^ = 0.02 [20]. Missing values were handled with listwise deletion in all analyses.

## Results

There were 258 patients who underwent resection for rectal carcinoma between January 2003 and December 2013. One hundred seven patients were excluded (Fig. 1). The remaining 151 patients had a mean age of 62 (SD, 11) years with a range from 24 to 90 years. Patients were divided into the following two groups: Group A (< 65 years) and Groups B (≥ 65 years). Group A included 79 patients, and Group B included 72 patients (Table 1). There was a higher number of patients with diabetes mellitus (*P* = 0.02), arterial hypertension (*P* = 0.022), and coronary heart disease (*P* = 0.002) in Group B compared to Group A. In the CKD stage, Group B showed a higher number of patients with CKD stage 2 and above relative to Group A (*P* < 0.001). No patients had a CKD stage 5 before undergoing LAR. SCr at T_0_ was higher in Group B (*P* = 0.04). Accordingly, the eGFR at T_0_ in group B was lower (*P* < 0.001). For tumor stage we found no differences between the groups (*P* = 0.18). There was no statistically significant difference between neoadjuvant treatment regimens between groups. However, fewer patients received adjuvant treatment in Group B compared with Group A (*P* = 0.001).

**Table 1:** Patients and tumor characteristics. The Chi-square test was used for analysis of categorical variables. The § indicates when Fisher's exact test was used. The Student’s *t*-test was performed for continuous variables. A *P*-value <0.05 (marked with *) was considered to be statistically significant

Variables	All (%)*n* = 151	Group A (%) *n* = 79	Group B (%)*n* = 72	Pearson’s Chi-Square(df)	Cramer’s V	*P*-value
Sex				0.47 (1)	0.06	0.49
Female	46 (30)	26 (33)	20 (28)			
Male	105 (70)	53 (67)	52 (72)			
ASA				3.91 (1)	0.16	0.05
≥3	65 (57)	28 (35)	37 (51)			
Comorbidities						
Diabetes mellitus	22 (15)	6 (8)	16 (22)	6.48 (1)	0.21	0.02*
Coronary heart disease	68 (45)	26 (33)	42 (58)	9.84 (1)	0.26	0.002*
Hypertension	57 (38)	23 (29)	34 (47)	5.26 (1)	0.19	0.022*
COPD	6 (4)	5 (6)	1 (1)	2.41 (1)	0.13	0.12
Renal function (CKD stage) at T_0_(for patients undergoing reversal)				27.7 (3)	0.49	<0.001*
G1	63 (54)	46 (77)	17 (30)			
G2	45 (39)	14 (23)	31 (55)			
G3	7 (6)	0	7 (13)			
G4	1 (.9)	0	1 (2)			
UICC stage n = 4 missing				6.26 (4)	0.21	0.18
0	19 (13)	11 (14)	8 (12)			
I	44 (30)	17 (22)	27 (39)			
II	18 (12)	12 (15)	6 (9)			
III	39 (27)	21 (27)	18 (26)			
IV	27 (18)	17 (22)	10 (15)			
Neoadjuvant Chemotherapy				0.60 (1)	0.06	0.44
Yes	95 (63)	52 (66)	43 (60)			
Neoadjuvant radiotherapy				1.37 (1)	0.10	0.24
Yes	92 (63)	51 (67)	41 (58)			
Adjuvant Chemotherapy				10.24 (1)	0.26	0.001*
Yes	60 (40)	41 (52)	19 (26)			
	All (mean, SD)	Group A (mean, SD)	Group B (mean, SD)	F-ratio		*P-*value
Age in years	62 (11)	54 (8)	71 (6)	243		<0.001*
Creatinine at T_0_(mg/dl)	0.86 (0.26)	0.80 (0.16)	0.93 (0.33)	8.48		0.04*
eGFR at T_0_ (ml/min/1.73m^2^)	88.38 (17.0)	97.14 (11.94)	78.87 (16.63)	48.75		<0.001*
Serum Potassium at T_0_mmol/l	4.17 (0.39)	4.18 (0.39)	4.17 (0.39)	0.03		0.88

ASA, American Society of Anesthesiologists; COPD, chronic obstructive pulmonary disease; UICC, Union for International Cancer Control; POD, postoperative day; df, the degree of freedom; eGFR, estimated glomerular filtration rate; SD, standard deviation

We found no differences between the groups for performing LAR except that more patients in Group A received a coloanal anastomosis (*P* = 0.03). Especially there were no statistically significant differences in postoperative complications (Table 2). 123 patients (82%) underwent reversal of DLI. We found no statistically significant differences were between both groups (Table 3). From all 151 patients, 14 patients (9.3%) were lost to follow-up.

**Table 2:** Characteristics of low anterior resection and short-term outcomes. The Chi-square test was used for analysis of categorical variables. The § indicates when Fisher's exact test was used. The Student’s *t*-test was performed for continuous variables. A *P*-value <0.05 (marked with *) was considered to be statistically significant

Variables	All (%) *n* = 151	Group A *n* = 79	Group B *n* = 72	Pearson’s Chi Square (df)	Cramer’s V	*P*-value
Operation				0.23 (2)	0.04	0.89
Open	126 (83)	67 (85)	59 (82)			
Laparoscopic	23 (15)	11 (14)	12 (17)			
Conversion	2 (1)	1 (1)	1 (1)			
Type of anastomosis				0.93 (3)	0.25	0.03*
End-end	45 (30)	18 (23)	27 (38)			
Side-end	60 (40)	30 (38)	30 (42)			
J-pouch	26 (17)	15 (19)	11 (15)			
Coloanal	20 (13)	16 (20)	4 (6)			
R1 resection	3 (2.0)	2 (3)	1 (1)	2.12 (2)	0.12	0.35§
CDC				0.10 (1)	0.03	0.76
≥ 3	34 (23)	17 (22)	17 (24)			
Complications						
Fascial dehiscence	2 (1)	2 (3)	0	1.85 (1)	0.11	0.50§
Surgical site infections	32 (21)	17 (22)	15 (21)	0.01 (1)	0.01	0.92
Deep vein thrombosis	1 (1)	1 (1)	0	0.92 (1)	0.08	0.52§
Lung embolism	1(1)	1 (1)	0	0.92 (1)	0.08	0.52§
Urinary tract infections	17 (11)	6 (8)	11 (16)	2.32 (1)	0.12	0.12
Pneumonia	6 (4)	1 (1)	5 (7)	3.18 (1)	0.15	0.10§
Paralytic ileus	9 (6)	5 (6)	4 (6)	0.04 (1)	0.02	0.84§
Anastomotic leakage	19 (13)	9 (11)	10 (14)	0.21 (1)	0.04	0.64
AKI (KDIGO)				5.14 (3)	0.21	0.16
No AKI	106(89)	56 (90)	50 (88)			
Stage I	9 (8)	6 (10)	3 (5)			
Stage II	2 (2)	0	2 (4)			
Stage III	2 (2)	0	2 (4)			
Reoperation	25 (17)	12 (15)	13 (18)	0.22 (1)	0.04	0.64
	All (mean, SD)	Group A (mean, SD)	Group B (mean, SD)	F-ratio		*P*-value
Duration of surgery (minutes)	240 (80)	245 (73)	234 (87)	0.61		0.44
Tumor distance from anal verge (centimeter)	8 (4)	8 (4)	8 (4)	0.05		0.83
Length of Hospital stay in days	17 (11)	16 (10)	18 (12)	0.90		0.35
Time to reversal of DLI in days	160 (136)	163 (149)	158 (122)	0.05		0.83
Follow up in months	56 (44)	62 (43)	50 (43)	2.43		0.12

*AKI *Acute Kidney Injury, *CDC *Clavien–Dindo classification, *ICU *Intensive care unit, *POD*, Postoperative day, *df *The degree of freedom, *KDIGO *Kidney disease: Improving global outcome, *SD *Standard deviation

**Table 3:** Characteristics of DLI closure. The Chi-square test was used for analysis of categorical variables. The § indicates when Fisher's exact test was used. The Student’s *t*-test was performed for continuous variables. A *P*-value <0.05 (marked with *) was considered to be statistically significant

Variables	All (%)	Group A	Group B	Pearson’s Chi Square (df)	Cramer’s V	*P*-value
Closure of DI				0.97 (2)	0.08	0.61
Yes	123 (82)	65 (83)	58 (82)			
Complications						
Surgical site infections	14 (9)	9 (11)	5 (7)	0.89 (1)	0.08	0.41
Diarrhea	23 (15)	13 (17)	10 (14)	0.19 (1)	0.04	0.66
Paralysis	11 (7)	5 (6)	6 (8)	0.22 (1)	0.04	0.64
Anastomotic leakage	2 (1)	0	2 (3)	2.22 (1)	0.12	0.23§
Late onset LAR anastomotic leakage	1 (1)	1 (1)	0	0.92 (1)	0.08	1§
CDC				3.7 (3)	0.16	0.30
3	6 (4)	1 (1)	5 (7)			
AKI (KDIGO)				4.80 (2)	0.21	0.10
No AKI	107 (93)	58 (97)	49 (91)			
Stage I	4 (4)	0	4 (7)			
Stage II	3 (23)	2 (3)	1 (2)			
Stage III	0	0	0			
Reasons for declining reversal (n=28)						
Patient’s preference	3 (11)	2 (14)	1 (7)	0.25 (1)	0.04	0.62§
Insufficient sphincter	5 (18)	2 (14)	3 (21)	1.23 (1)	0.09	0.35
Tumor progression	11 (40)	7 (50)	4 (29)	0.61 (1)	0.06	0.44
Death before reversal	2 (7)	1 (7)	1 (7)	0.004 (1)	0.005	0.95§
Unknown reasons	6 (21)	2 (14)	4 (29)	0.90 (1)	0.07	0.43§
	All (mean, SD)	Group A (mean, SD)	Group B (mean, SD)	F-ratio		*P*-value
Duration of surgery (minutes)	85 (53)	82 (41)	88 (63)	0.53		0.47
Length of Hospital stay in days	10 (14)	9 (16)	10 (11)	0.09		0.77
Time to reversal of loop ileostomy in days	160 (136)	163 (149)	158 (122)	0.05		0.83

*AKI *Acute Kidney Injury, *CDC *Clavien–Dindo classification, *ICU *Intensive care unit, *POD *Postoperative day, *df *The degree of freedom, *KDIGO *Kidney disease: Improving global outcome, *SD *Standard deviation

### SCr, eGFR and CKD at T_0_

At T_0_, Group B had more patients with CKD ≥2 (*P* < 0.001), higher SCr levels (*P* = 0.04), and lower eGFR (*P* < 0.001) compared to Group A.

### Acute kidney injury after LAR and DLI reversal 

After LAR 13 patients (11%) developed AKI: stage I, 9 patients (7.6%); stage II, 2 patients (1.7%); and stage III, 2 patients (1.7%). No patient had to undergo hemodialysis after the surgery and all AKI were treated with additional fluid infusions and diuretic medications.

Seven patients (6.1%) developed an AKI stage I or II after DLI reversal. No patient developed a stage III AKI. There was no statistically significant difference in developing AKI between the age groups neither for LAR nor DLI reversal.

All patients in Group A had a renal function according to CKD in stage G1 or G2 at T_0_ and T_1_: stage G1, T_0_: 46 (78%), T_1_: 24 (69%); stage G2, T_0_: 14 (23%), T_1_: 11 (31%).

Patients in Group B had a renal function according to CKD varying from G1 to G4 at T_0_ and T_1_: stage G1, T_0_: 17 (30%), T_1_: 7 (24%); stage G2, T_0_: 31 (55%), T_1_: 12 (41%); stage G3, T_0_: 7 (13%), T_1_: 9 (31%); stage G4, T_0_: 1 (2%), T_1_: 1 (3%).

No patient had a CKD stage G5. There were no statistically significant differences within the groups.

### Risk factors for persistent renal impairment 3 months after DLI reversal 

Multivariate linear regression analyses for factors influencing SCr and eGFR 3 months after DLI reversal was performed.

Age and SCr at T_0_ were able to predict increase of SCr at T_1_ (F(14,19) = 6.06; *p *< 0.001). The R² for the overall model was 0.82 (adjusted R² = 0.68).

For decreasing of eGFR at T_1_ age and eGFR at T0 were shown as predictors (F(14,51) = 3.33; *p* = 0.001). The R² for the overall model was 0.48 (adjusted R² = 0.33). Results are also shown in Table 4.

**Table 4:** Multivariate linear regression analysis of factors related to absolute changes in serum creatinine and eGFR, comparing the level 3 months after DLI reversal (T_1_) and preoperative baseline (T_0_)

	**Serum Creatinine**				**eGFR**			
	Multivariate regression coefficient	Beta	95% CI	*p* value	Multivariate regression coefficient	Beta	95% CI	*p* value
**Patient-related factors**								
Age	0.009	0.313	0.002 – 0.015	**0.009**	-0.617	-0.304	-1.134 - -0.100	**0.020**
Sex (male)	-0.034	-0.054	-0.191 – 0.122	0.664	0.778	0.017	-8.021 – 9.576	0.860
Diabetes mellitus	0.038	0.048	-0.156 – 0.232	0.697	-4.358	-0.077	-15.960 – 7.244	0.454
Hypertension	-0.010	-0.016	-0.166 – 0.145	0.895	-1.962	-0.042	-11.244 – 7.320	0.673
COPD	-0.030	-0.021	-0.368 – 0.307	0.858	6.940	0.066	-13.170 – 27.050	0.491
ASA Score	0.057	0.147	-0.368 – 0.307	0.236	-3.300	-0.118	-9.018 – 2.417	0.252
Neoadjuvant Chemotherapy	0.097	0.139	-0.074 – 0.268	0.260	-6.607	-0.132	-16.741 – 3.528	0.196
Adjuvant Chemotherapy	-0.026	-0.042	-0.175 – 0.123	0.726	-1.592	-0.036	-10.720 - 7536	0.728
Serum creatinine at T_0_	0.754	0.494	0.320 – 1.187	**0.001**				
eGFR at T_0_					0.775	0.582	0.421 – 1.130	**<0.001**
**Procedure-related factors**								
CDC (LAR)	-0.088	-0.115	-0.280 – 0.103	0.358	4.475	0.081	-7.021 – 15.971	0.438
Blood transfusion (LAR)	-0.040	-0.061	-0.208 – 0.128	0.633	0.892	0.019	-9.257 – 11.041	0.861
Operation time (LAR)	0	-0.063	-0.001 – 0.001	0.590	0.017	0.058	-0.040 – 0.073	0.554
CDC (DLI)	-0.031	-0.100	-0.103 – 0.041	0.391	1.022	0.046	-3.301 – 5.345	0.637
Time to DLI reversal	0	0.139	0.000 – 0.001	0.227	-0.022	-0.090	-0.068 – 0.025	0.351

*ASA *American Society of Anesthesiologists, *COPD *Chronic obstructive pulmonary disease, *CDC *Clavien–Dindo classification, *ASA,  DLI *Diverting loop ileostomy, *eGFR *Estimated glomerular filtration rate, *LAR *Low anterior resection

## Discussion

This study investigated if older patients are prone to a decline in kidney function following the creation of DLI after LAR for rectal cancer. We were able to show older age, increased SCr and decreased eGFR before LAR to be significant prognostic factors for a decline in kidney function 3 months after DLI reversal.

The need for DLI creation along with LAR remains controversial. While DLI creation seems to protect patients from the life-threatening complications of anastomotic leakage after LAR it can also lead to known complications such as dehydration, and worsening of preexisting kidney disease [21, 22]. Other studies also suggested that complications related to DLI creation can delay adjuvant chemotherapy.

The main concern after DLI creation is kidney dysfunction resulting from high-output stomata associated with dehydration. Possible long-term consequences of dehydration resulting from high-output DLI after LAR have been described by Fielding et al. who showed that DLI significantly increased the risk of new onset or worsening CKD [23]. Our study found that with increasing age and preoperative reduced eGFR the risk for reduced eGFR 3 months after DLI reversal is statistically significant higher. Even if the results of this study cannot be used to determine the long-term development of kidney function in this cohort, other studies have shown that even a short-term decline in kidney function can lead to a long-term renal dysfunction and a higher risk for myocardial infarction [24, 25]. Reasons, why increasing age could lead to a long-term renal dysfunction are multifactorial. Older patients are likely to have a higher rate of diabetes, chronic kidney failure and cardiac diseases, followed by a polypharmacy with protentional kidney toxic medications, such as diuretics and ACE inhibitors [18, 23]. This could lead to a preexisting reduction of eGFR [9, 22]. For our patient’s cohort we did not find any other predicating factors for impairment of renal function. Reasons for this could be the very homogeneous study population, as only patients undergoing elective surgery for rectal cancer were included. Nevertheless, due to the non-inclusion of patients medication in the multivariate linear regression analyses, the study may be biased.

On the other hand, we showed in this study, that no patient had to undergo postoperative hemodialysis due to kidney failure. But also if no patient developed CKD stage 5, even a slight or temporary decrease in renal function is known to promote the development and progression of chronic kidney failure [24]. This could lead to the development of chronic renal impairment or worsening of this pre-existing condition.

Some authors suggest the creation of a loop colostomy instead to avoid high-output stoma and dehydration. An actual meta-analysis by Gavriilidis et al. compared loop transverse colostomy and loop ileostomy [26]. They showed that patients with ileostomy tend to have a significantly higher rate of complications due to high-output stoma. Patients with colostomy developed more complications after reversal surgery, but analysis of the overall complications did not favor ileostomy or colostomy. Some other studies had a similar conclusion [27, 28].

This study has some limitations. First, a retrospective database from a single center institution was used for the analyses along with a small cohort. The temporal distance between time points T_0_ and T_1_, differ variably between the patients, as DLI reversal was performed at different time points. It was also not possible to retrospectively measure proteinuria, which is necessary for a complete assessment of kidney function based on the KDIGO-guidelines[14]; thus, the true incidence of chronic kidney failure might be underscored in this analysis. One shortcoming of our study is the fact, that we only were able to analyze SCr and eGFR after LAR and 3 months after DLI. Whether a protective DLI creation has a long-term impact on kidney function in older patients, an further analysis, e.g. one year after DLI reversal, would be necessary.

For multivariate linear regression analysis multicollinearity is a problem, as patients with a higher age are more likely to have an already impaired eGFR and increased SCr.

One important strength of this study is the choice of only elective patients with rectal cancer. There is no bias in this study population due to included emergency surgeries of patients with inflammatory bowel disease. Age is a major concern for surgeons when planning surgical procedures in cancer patients. We showed here that the choice for DLI leads to a significantly impaired kidney function for older patients even after stoma reversal.

## Conclusions

In summary, DLI creation along with LAR for rectal cancer may result in a long-term reduction of eGFR in older patients. Of course, surgeons should consider the above-mentioned negative impact of a loop ileostomy when planning a diverting ostomy for LAR in older patients while carefully monitoring kidney function.

## Data Availability

The datasets generated and analyzed during the current study are not publicly available due to the Ethics Committee restrictions but are available from the corresponding author on reasonable request.

## Abbreviations

AKI: Akute kidney injury
ASA: American Society of Anesthesiologists
CDC: Clavien–Dindo classification system
CKD: Chronic kidney disease
CKI-EPI: Chronic Kidney Disease Epidemiology Study
COPD: Chronic obstructive pulmonary disease
df: Degree of freedom
DLI: Diverting loop ileostomy
eGFR: Estimated glomerular filtration rate
FOLFIRI: folinic acid, 5-fluorouracil, and irinotecan
FOLFOX: 5-fluorouracil or oxaliplatin, folinic acid, and 5-fluorouracil
ICU: Intensive Care Unit
KDIGO: Kidney Disease Improving Global Outcomes guidelines
LAR: Low anterior resection
POD: Postoperative Day
RRT: Renal replacement therapy
SCr: Serum creatinine
SD: Standard deviation
SPSS: Statistical Package for the Social Sciences
TME: Total mesorectal excisions
UICC: Union for International Cancer Control
